# The Use of Low-Dose Methadone as Add-On to Ongoing Opioid Treatment in Palliative Cancer Care—An Underrated Treatment?

**DOI:** 10.3390/life12050679

**Published:** 2022-05-03

**Authors:** Per Fürst

**Affiliations:** 1 Department of Oncology-Pathology, Karolinska Institutet, 171 64 Stockholm, Sweden; per.furst@ki.se; 2 Palliative Medicine, Stockholms Sjukhem Foundation, SE-112 19 Stockholm, Sweden

**Keywords:** methadone, pain, palliative, cancer, opioid

## Abstract

The aim of this review is to summarize the current knowledge of low-dose methadone treatment in palliative cancer care. In Sweden, methadone is quite common in specialized palliative care, where almost a tenth of patients are prescribed this drug. Negative attitudes towards methadone do not seem to prevent it from being used for pain management, and by starting with low doses and then increasing slowly and gradually, methadone can apparently be introduced safely. It is still uncertain whether methadone has a better analgesic effect than other opioids. However, for pain relief in cancer patients with severe and complex cancer-related pain, NMDA receptor inhibition with methadone may, in selected cases, be an attractive alternative, especially in the form of low-dose supplements to other ongoing opioids. Due to long half-life and complex metabolism, the use of methadone requires an experienced physician and solid follow-up. Continuous administration of opioids, including low-dose methadone, has been proven effective and safe in reducing pain in dying patients without increasing the risk of confusion, regardless of age.

## 1. Introduction

It is common for patients with advanced cancer to have pain [1]. Fortunately, pain treatment has improved in recent years and for most patients with advanced cancer, pain treatment works well [1,2,3,4,5]. The analgesic therapy is always tried to be tailored according to the mechanism causing the pain. Sometimes simple medications such as paracetamol, naproxen or ibuprofen are enough, even for pain from bone metastases. However, for many patients it is also necessary to receive treatment with strong opioids [3]. With opioids, most people become pain-free without getting sedated from the medication [5].

Morphine and other strong opioids do work well for many pain mechanisms, but not all [1]. Sometimes, this is because there are different types of pain present at the same time, with different underlying mechanisms [1]. In neuropathic and mixed nociceptive-neuropathic pain, the analgesic effect of opioids varies and is often inadequate, even with fairly high doses of opioids.

Treatment of neuropathic pain in cancer differs in part from treatment of neuropathic pain in non-cancerous conditions. It is common for opioids to be combined with tricyclic antidepressants, gabapentin or pregabalin and/or serotonin-noradrenaline reuptake inhibitors. In cases of inflammatory components, steroids and non-steroidal anti-inflammatory drugs are also often used [6,7].

Although not an official designation, the term complex pain is used in this text to qualify pain that is refractory or partially refractory to first-line treatments and that may involve both nociceptive and neuropathic pain components, and where central sensitization may be involved [8,9,10]. Central sensitization means that an accelerated state of pain is caused even though there is no threatening or progressive tissue damage. The International Association for the Study of Pain (IASP) defines central sensitization as an increased sensitivity of nociceptive neurons in the central nervous system to their normal or subthreshold afferent inputs [11,12,13,14]. Simply put, it means that the nervous system has become hypersensitive. Pain impulses that would normally have been slowed down before they reached the brain now have a free flow and can cause severe pain conditions where ordinary opioids are not enough.

Central sensitization is associated with decreased opioid sensitivity and is mediated at least in part via activation of the N-methyl-D-aspartate (NMDA) receptor [15,16]. NMDA receptors are known to be involved in the development of allodynia, hyperalgesia, opioid tolerance and opioid-resistant neuropathic pain [17]. If that receptor can be inhibited, the pain can be better relieved. There are drugs that inhibit NMDA receptors. They can reduce the sensitization of the nervous system and make the pain more sensitive to opioids again. One such drug is methadone, which is perhaps best known for its use in heroin withdrawal.

The first part of this review will summarize the current knowledge of low-dose methadone as an add-on to ongoing opioid treatment in palliative cancer care.

## 2. Methadone as an Analgesic in Cancer-Related Pain

Methadone is an opioid that acts as an analgesic via mu-receptors, but in addition, it also inhibits serotonin and norepinephrine uptake and NMDA receptors to some extent [15,18,19,20,21]. Therefore, it may be useful for relieving complex cancer pain. Unfortunately, methadone also has some disadvantages, including a long half-life varying from 5 to 130 h with a mean of 20–35 h [20,22]. In addition, the rate at which the liver metabolizes methadone can vary greatly between different individuals and within the same individual at different time-points, making methadone quite difficult to control. The half-life is shorter for patients using methadone continuously, probably due to autoinduction of methadone’s metabolism [22].

Via the Cytochrome P450 (CYP450) enzyme system, methadone affects the liver metabolism of other drugs [22]. Drugs that inhibit CYP450 will reduce methadone’s metabolism, resulting in increased methadone levels. CYP450 inducers will have the opposite effect. The analgesic effect becomes more difficult to predict and therefore considerable clinical experience is required to prescribe and manage a methadone treatment in a safe way. Examples of drugs that may increase methadone concentration include antibiotics (ciprofloxacin, erythromycin, trimethoprim), antidepressants (citalopram, fluoxetine, sertraline, paroxetine), antifungals (fluconazole, ketoconazole), benzodiazepines (midazolam, diazepam, alprazolam), cardiac drugs (verapamil, nifedipine, amiodarone), NSAIDs (celecoxib), neuroleptics (haloperidol), PPIs (omeprazole, esomeprazole, lansoprazole), TCAs (amitriptyline) and cannabinoids. Medications that may decrease methadone concentration comprise anticonvulsants (phenytoin, carbamazepine, phenobarbital), antiretroviral agents (nevirapine, rifampin) and St John’s Wort [22,23].

Because methadone seems to have such a good effect against certain more severe conditions with cancer pain, it has of course continued to be used sometimes, even though it can be complicated. Most often, morphine has been replaced with methadone straight away and then the risks and any problems that have arisen have been dealt with [24].

In cancer pain, methadone is currently recommended as a second-line opioid [25]. Methadone is currently most used when switching from another opioid when trying to improve the balance between analgesic effect and side effects [25]. The combined properties of methadone with, among others, effect on µ-receptors and NMDA receptors without active metabolites means that methadone has the potential to contribute to a good pain-relieving effect with fewer adverse effects than other opioids [26]. In reviews, it was described that high doses of methadone in cancer-related pain have a similar or even better effect compared to both morphine and transdermal fentanyl [25,27,28,29,30,31,32]. At the same time a higher frequency of somnolence was seen, and patients dropped out of the studies, which possibly may be related to adverse effects [25,27,28,29,30,31,32]. It is common that the dose of methadone does not need to be escalated over time [25,29,33,34].

Evidence of analgesic effect in chronic neuropathic pain has been reported [35]. However, in the 2017 Cochrane reviews dealing with methadone for cancer pain and neuropathic pain in adults, no conclusions could be drawn about differences in analgesic effect or safety when comparing methadone with placebo, other opioids or other treatments [36]. However, the studies reviewed were relatively small and of rather low quality. There is also more recent evidence that methadone may also reduce neuropathic cancer pain [37].

### Low-Dose Methadone as an Add-On to Regular Opioid Therapy

In those cases where a sufficient analgesic effect has still not been achieved, despite increasing the opioid doses, the practice for a long time has been to attempt a complete rotation over to methadone and only use other opioids for breakthrough pain [24]. In 2004, Mercadante et al. described for the first time an “opioid semi-switching” regimen where 14 patients with cancer-related pain who had at least doubled their opioid doses in the past week had fentanyl or methadone added in an initial dose of 20% of the ongoing opioid regimen [38]. The regimen seemed successful, with stopped successive opioid escalation and improved analgesic effect of the ongoing opioid treatment [38].

It then took a few years until two letters were published regarding adjunctive therapy with low-dose methadone, one from McKenna, in 2011, and one from Haughey et al., in 2012 [39,40]. The letters described ten and three cases, respectively. In these letters, an interesting idea was presented: methadone could be used for pain in a safe and successful way by combining it with another ongoing opioid, if it was introduced gently in a low dose. In this way, one could benefit from the NMDA receptor antagonism, without having to make a complete switch to methadone in the usual way, and at the same time be able to minimize the risks associated with methadone’s complex pharmacology.

Above all, it seemed that this simpler method could be of value for methadone use in palliative care, where severe pain is also often treated in outpatient care [39].

A few smaller retrospective cohort studies have been performed regarding adjunctive therapy with low-dose methadone [41,42]. These usually describe supplements with 2.5–5 mg b.i.d. of oral methadone that is safely combined with a regular opioid to achieve improved analgesia in complex cancer-related pain. Methadone doses are usually stabilized at about 10–15 mg per day within a week. In 49–75% of cases, improved pain control is reported. Chary described the 2019 pain-relieving regimen with so-called ultra-low additional doses of methadone that started with 1 mg daily and then the daily dose increasing by 1 mg once a week. The maximum dose was 20 mg methadone daily and analgesia onset was slow [43]. Courtemanche et al. described how they observed a plateau in response to an increasing dose of methadone [42]. This may indicate that if patients receive a good analgesic effect already from a low dose of methadone, they may not experience a further reduction in pain intensity even if the methadone dose is increased, compared with if the dose is kept unchanged. This low-dose add-on methadone therapy has become increasingly popular. Low-dose methadone usually refers to methadone doses that do not exceed 20 mg per day [39,40,41,42,43] (Table 1). 

## 3. Four Studies on Methadone Use in Palliative Care

In addition to describing the hitherto sparse studies in the field of low-dose methadone as an add-on to another ongoing opioid for cancer-related pain, presented here is a comprehensive report on four more recent studies that were designed and performed to supplement the knowledge on the subject. The studies were included in a thesis from Karolinska Institutet in December 2020 (Table 2).

Conducting clinical trials in end-of-life patients is often challenging. On the one hand, all functions deteriorate rapidly, and on the other hand, quite naturally in such a situation, patients have other priorities than participating in research studies. Not least the relatives and even the staff, sometimes subconsciously, try to protect the patients from being involved, without even asking patients what they want, a phenomenon called gatekeeping. The dropout can be large.

To perform placebo-controlled studies is often not possible due to ethical reasons. The four studies described below on methadone in palliative care are observational studies and one qualitative study.

The aim of the first study was to investigate whether a low-dose add-on of methadone to another ongoing opioid therapy could contribute to pain relief in dying patients with complex cancer pain, as well as examining the possible adverse effects in the form of sedation, delirium and/or respiratory depression [45]. The medical records from all patients who had been cared for at the Stockholms Sjukhem palliative clinic during the years 2006–2013 and who had been prescribed methadone were studied. Eighty patients were found who, in addition to their usual opioid, had been given tablets with a low dose of methadone for pain. Overall, most (80%) had experienced significantly less pain and needed less morphine during the first week after methadone was initiated. The side effects seen were none other than those commonly seen with morphine.

The second study aimed at investigating the use of methadone in specialized palliative care in Sweden and specifically explore the frequency of use, indications, doses, opioid combinations and adverse effects when using low dose methadone in combination with other opioids at the end-of-life [46]. This was explored by asking palliative care units to fill in an additional methadone survey that appeared after the usual registration of deceased patients in the Swedish Registry of Palliative Care. The 60 participating palliative care units reported data on 4780 patients, of whom 410 (8.6%) had been prescribed methadone while in palliative care, in 96% of cases as a low-dose add-on to other opioids and most often against cancer pain. In a third of the cases, the prescription was made when the patient was cared for at home. In addition, it was reported that the vast majority had received better pain relief due to the use of methadone (94%). The side effects were few and appeared rarely.

The third study was slightly different [47]. It aimed at broadening and deepening the understanding of attitudes about, potential significance of and practical aspects regarding the use of methadone for pain in specialized palliative care. In this study, a total of 30 physicians in palliative care and pain care were interviewed about their experiences with low-dose methadone as an add-on. The physicians were selected to provide as broad a knowledge base as possible through variation in experience, age, sex, and workplace. There was a suspicion that it might be difficult to convince patients to use methadone if it might be associated with heroin abuse. Admittedly, many made that connection, but it was not described at all as an obstacle in everyday life to use methadone for pain. The physicians described how methadone was used. They thought it worked best partly in patients who had needed morphine or other opioids for pain but where it no longer helped so well, and partly in cases with suspected central sensitization and difficult-to-treat cancer pain in the spine or pelvis. Typical diagnoses they mentioned as extra appropriate to methadone were skeletal metastases of prostate, breast, or kidney cancer but also pancreatic cancer and sarcoma.

In practical terms, the doctors described how they, by starting with low doses of methadone and then increasing the doses gradually, could achieve both a good pain-relieving effect and at the same time avoid serious side effects, even when used in home care. If methadone started to cause a good analgesic effect within a few days, they often reduced the dose of the already ongoing opioid by 25–30% to reduce the risk of opioid overdose. The analgesic effect was described as able to last for at least weeks or months. In most cases, this was a sufficiently long time in specialized palliative care.

In the fourth study, the aim was to prospectively study analgesic and adverse effects when prescribing subcutaneous infusion via an ambulatory infusion pump to imminently dying patients in specialized palliative care with a special focus on add-on low-dose methadone [48]. The daily symptoms of 93 dying patients who were prescribed continuous subcutaneous drug administration were followed. The reason for transition to subcutaneous administration was usually increasing difficulty in swallowing tablets due to deteriorating general condition at the end of life or the need for better pain relief. Survival was on average four days (median). Pain relief was significantly improved in patients when the subcutaneous administration started without an increase in the incidence of delirium, not even when comparing patients older or younger than 75 years.

Another question was whether low-dose methadone for pain could be continued even in the syringe driver at the end of life. It turned out that it went well, and that methadone was used mainly for patients who were initially judged to have very severe pain, without any serious side effects.

The starting dose of methadone was reported in all studies to be a median of 5 mg per day, which was often increased to 10 mg and a maximum of 20 mg per day. The add-on therapy with low-dose methadone was usually given divided into two doses per day.

## 4. Discussion

For most patients with cancer-related pain, treatment with strong opioids provides adequate pain relief. However, regular opioids are not enough in all cancer pain situations. Due to the underlying pain mechanism, different types of pain require different types of additional treatment, for example, in case of severe mixed nociceptive and neuropathic pain or when the opioid doses have had to be increased rapidly without giving much better pain relief. These are situations where central sensitization of the nervous system can be suspected to contribute to a more complex pain situation. In some selected patients, NMDA receptor antagonists seem to be able to reduce the effects of central sensitization [49,50]. In these situations, the opioid methadone is sometimes successfully used.

Methadone is an opioid and thus has a µ-receptor effect that is comparable to, and not better than, other opioids in situations with common cancer pain. In those situations, a low dose of opioid is usually enough for good analgesia [25,27,29]. In cases where methadone is assessed the appropriate drug and a complete and potentially complicated conversion to methadone as the only opioid is planned, an alternative method with the addition of a low dose of methadone to the already ongoing opioid treatment has been described. This is a method applied by several specialized palliative care units in Sweden, where, during the 12-month study period, methadone for pain was prescribed to 8.6% of the patients who were cared for in the participating specialized palliative care units.

In the described studies, the indication for prescribing low-dose methadone in addition to another opioid in about 70–80% of cases was either assessed complex pain, that is, pain with difficult-to-treat mixed nociceptive and neuropathic components, or only neuropathic pain [45,46,48]. This was also supported in the interviews with physicians [47]. They described that, in addition to existential pain and death anxiety, pain with mixed components is often the most complex and difficult to alleviate in specialized palliative care, but that low-dose add-on of methadone may in these cases be a way to achieve better pain relief fast. The effect could then sometimes be unexpectedly good.

Compared with existing literature, there were relatively high proportions of successful treatment [41,42,43]. A probable reason behind this is that the studies were descriptive in nature and were not designed to find out which patients benefited most from low-dose methadone.

A probably important part of the explanation for which patients receive a good effect from the treatment regimen is the initial selection of patients. The fact that there was a larger proportion of patients with assessed improved analgesic effect compared with previous studies may thus be since not only pain intensity was in focus. Rather, the focus was on pain analysis and mechanisms behind the pain. This was best seen in the interviews with doctors who described how they found that patients who responded best to methadone were those who had had pain for a long time and where increasing opioid doses had not provided better pain relief.

The interviewed physicians themselves used different explanatory models when thinking about the underlying mechanisms and mentioned concepts such as “exhausted pain system”, “opioid tolerance” and “central sensitization”. They also gave examples of what types of pain could be thought to respond to methadone. Recurrent pain from skeletal metastases and pathological fractures and pain from the spine or pelvis were mentioned. Cancer of the pancreas, breast, prostate, kidney, and sarcoma were mentioned as specific diagnoses. As far as can be found in the existing literature, there are no previous descriptions of which patients could potentially benefit most from treatment with low-dose add-on of methadone, apart from the occurrence of severe pain [42].

Central sensitization was thus mentioned as a possible explanation for the onset of more complex pain. It is known that drugs with NMDA inhibitory effect, such as ketamine or 3-(2-carboxypiperazin-4-yl) propyl-1-phosphonic acid (CPP), can reduce the effects of central sensitization. This can provide better pain relief for difficult-to-treat chronic pain. In the case of cancer pain, there is no unequivocal evidence, which may be partly due to the difficulties in studying those patients [49,50,51,52]. Interestingly, it seems that treatment with ketamine for a short period of time improves the analgesic effect better than lower doses for longer periods [50]. As described, but of uncertain importance, methadone has both an µ-receptor inhibitory effect, serotonin and norepinephrine uptake inhibitory effect, and some effect on NMDA receptors [15,18,20]. Nerve cells seem to be able to fine-tune the different subunits in NMDA receptors and thus change their properties [53]. This could mean that subtype-specific antagonists for NMDA receptors may be a possible, not yet established, explanation behind the different effects that can be seen between different NMDA-inhibitory drugs [54].

It is well known from the concept of multimodal analgesia that there may be synergistic effect between two or more analgesic drugs [55]. Combinations may, for instance, include opioids, alpha-2 agonists, NMDA receptor antagonists, gabapentinoids, dexamethasone, non-steroidal anti-inflammatory drugs (NSAIDs), acetaminophen, and duloxetine [55]. However, previous knowledge and these recent findings combined do not make it unreasonable to assume that methadone can have an effect on central sensitization. However, the duration of the effect when adding a low dose of methadone is unclear, and in the available literature, the effect duration is described for between one week and up to at least several months [41,42,43]. Additionally, in the qualitative interview study, some of the physicians described how they saw improved pain relief for weeks to months after starting low-dose add-on methadone. It was even described that sometimes the analgesic effect could be seen even after the adjunctive treatment with low-dose methadone had ended and only the original regular opioid remained.

One could imagine an example of a situation with a patient who had an ongoing analgesic treatment with methadone as the only opioid, which is not as a low-dose add-on, and where methadone treatment was suddenly discontinued. The pain would then return quickly. Thus, a plausible explanation for why pain does not increase when an already successful treatment with low-dose methadone is discontinued could be that the NMDA-inhibitory, or other, effect of the low-dose methadone had already given a reduced central sensitization and thus “wind-down”.

The subject continues to be studied. A recent smaller randomized controlled trial found that a low dose of methadone in combination with morphine provided faster pain control in patients with cancer-related pain, as compared with morphine alone and without more adverse effects [44]. Interestingly, it was reported in this study that the increased analgesic effect of low-dose methadone appeared to be present for a limited period of a few weeks [44]. Additionally, Mercadante et al., recently prospectively assessed introduction of low-dose methadone in patients receiving low doses of opioids or none, and found significant reduction in pain intensity, limited adverse effects and minimal opioid-induced tolerance [34]. Improved pain control was still seen after two months. They concluded that this first-line regimen seems easier to use than opioid switching, for which high experience is needed [34].

## 5. Conclusions

Taken together, the studies give us pieces in a puzzle that contribute to the bigger picture. It is still uncertain whether methadone has a better analgesic effect than other opioids. However, the most important thing for achieving better pain relief is to identify the various mechanisms behind a complex pain and address them. Adequate medication or other appropriate action is then selected based on the identified pain mechanisms. For pain relief in advanced patients with severe and complex cancer-related pain, treatment with methadone may, in selected cases, be an attractive alternative to take advantage of methadone’s unique properties, especially in the form of low-dose supplements to other ongoing opioids. It seems to be both a simple and safe way to initiate and use methadone but requires good knowledge, experience, and follow-up. Further research on this topic would be of value, not least in the form of randomized controlled trials.

## Figures and Tables

**Table 1:** Characteristics of significant reports on low-dose methadone in combination with another ongoing regular opioid for cancer-related pain.

Author	Ref.	Design	*N*	Initial Methadone Dose (Mean mg/Day)	Maximum Dose of Methadone (Mean mg/Day)	Regular Opioids	Regular Opioid Doses	Analgesic Response	Follow-Up	Severe Adverse Effects
Mercadante, 2004	[38]	Prospective open label	14	12.2	13.3	Mo	Decreased escalation	Significantly improved	Five weeks	No significant increase
McKenna, 2011	[39]	Retrospective case report	10	10 (median)	20 (median)	Ox, Mo	Decreased	Very effective	Up to 6 months	No reported
Haughey, 2012	[40]	Retrospective case report	3	3.3	16.7	Ox, Mo	Decreased	Improved	15 days	Opioid toxicity disappeared
Wallace, 2013	[41]	Retrospective observational	20	4.4	15.5	Mo, Ox, Fe, Hy	Stable	Improved	1 month	Prolonged QTc in two cases
Courtemanche, 2016	[42]	Retrospective observational	146	3 (median)	9 (median)	Mo, Ox, Fe, Hy	Stable	49% of cases had ≥30% reduction	60 days	Opioid overdose in one case
Chary, 2020	[43]	Retrospective observational	35	1	9 (median)	Mo, Ox, Fe, Hy	Sign. reduced	Sign. improved in 68%	55.6 weeks (mean)	No reported
Duarte, 2021	[44]	Randomized control	41	5	5	Mo	Stable	Sign. improved after 2 weeks only	3 months	No reported
Mercadante, 2022	[34]	Prospective open label (subgroup)	20	9	10.5	Mo, Ox, Fe, Hy, Ta, Bu	Stable	Sign. improved	2 months	No reported

Ref., bibliographical reference number; Mo, Morphine; Ox, Oxycodone; Hy, Hydromorphone; Fe, Transdermal fentanyl; Ta, Tapentadol; Bu, Buprenorphine; QTc, QT-interval on the ECG corrected for heart rate; Sign., Significantly.

**Table 2:** The four studies reported in Section 3.

	Ref.	Design	Data Source	Outcome
Study I	[45]	Retrospective observational	Medical records	Intensity of pain, opioid doses incl. methadone, adverse effects.
Study II	[46]	Retrospective observational	Swedish Registry of Palliative Care	Prevalence, indications and reported effects, opioid doses incl. methadone, adverse effects.
Study III	[47]	Qualitative interview study	Physicians in specialized palliative care	Attitudes to, indications for and practical use of methadone.
Study IV	[48]	Prospective observational	Patients in specialized palliative care	Level of pain and other symptoms, opioid doses incl. methadone, adverse effects.

Ref., bibliographical reference number.

## Data Availability

Not applicable.
